# Rootstock Sub-Optimal Temperature Tolerance Determines Transcriptomic Responses after Long-Term Root Cooling in Rootstocks and Scions of Grafted Tomato Plants

**DOI:** 10.3389/fpls.2017.00911

**Published:** 2017-06-08

**Authors:** Georgia Ntatsi, Dimitrios Savvas, Vassilis Papasotiropoulos, Anastasios Katsileros, Rita M. Zrenner, Dirk K. Hincha, Ellen Zuther, Dietmar Schwarz

**Affiliations:** ^1^Laboratory of Vegetable Crops, Department of Crop Science, Agricultural University of AthensAthens, Greece; ^2^Department of Agricultural Technology, Technological Education Institute of Western GreeceAmaliada, Greece; ^3^Laboratory of Plant Breeding and Biometry, Department of Crop Science, Agricultural University of AthensAthens, Greece; ^4^Leibniz Institute of Vegetable and Ornamental CropsGroßbeeren, Germany; ^5^Central Infrastructure Group Genomics and Transcript Profiling, Max-Planck-Institute of Molecular Plant PhysiologyPotsdam, Germany

**Keywords:** grafting, phytohormones, abiotic stress, microarrays, physiology, *Solanum lycopersicum*, *Solanum habrochaites*

## Abstract

Grafting of elite cultivars onto tolerant rootstocks is an advanced strategy to increase tomato tolerance to sub-optimal temperature. However, a detailed understanding of adaptive mechanisms to sub-optimal temperature in rootstocks and scions of grafting combinations on a physiological and molecular level is lacking. Here, the commercial cultivar Kommeet was grafted either onto ‘Moneymaker’ (sensitive) or onto the line accession LA 1777 of *Solanum habrochaites* (tolerant). Grafted plants were grown in NFT-system at either optimal (25°C) or sub-optimal (15°C) temperatures in the root environment with optimal air temperature (25°C) for 22 days. Grafting onto the differently tolerant rootstocks caused differences in shoot fresh and dry weight, total leaf area and dry matter content of roots, in stomatal conductance and intercellular CO_2_ and guaiacol peroxidase activity but not in net photosynthesis, sugar, starch and amino acid content, lipid peroxidation and antioxidant enzyme activity. In leaves, comparative transcriptome analysis identified 361 differentially expressed genes (DEG) responding to sub-optimal root temperature when ‘Kommeet’ was grafted onto the sensitive but no when grafted onto the tolerant rootstock. 1509 and 2036 DEG responding to sub-optimal temperature were identified in LA 1777 and ‘Moneymaker’ rootstocks, respectively. In tolerant rootstocks down-regulated genes were enriched in main stress-responsive functional categories and up-regulated genes in cellulose synthesis suggesting that cellulose synthesis may be one of the main adaptation mechanisms to long-term sub-optimal temperature. Down-regulated genes of the sensitive rootstock showed a similar response, but functional categories of up-regulated genes pointed to induced stress responses. Rootstocks of the sensitive cultivar Moneymaker showed in addition an enrichment of up-regulated genes in the functional categories fatty acid desaturation, phenylpropanoids, biotic stress, cytochrome P450 and protein degradation, indicating that the sensitive cultivar showed more transcriptional adaptation to low temperature than the tolerant cultivar that did not show these changes. Mainly defense-related genes were highly differentially expressed between the tolerant and sensitive rootstock genotypes under sub-optimal temperature in the root environment. These results provide new insights into the molecular mechanisms of long-term sub-optimal temperature tolerance of tomato.

## Introduction

A commonly encountered abiotic stress for cold-sensitive vegetables that restricts their yield potential is the exposure to sub-optimal temperatures, i.e., cultivation above the minimum growth temperature range of 8–12°C instead of at the optimum temperature range of 18–27°C (Criddle et al., 1997; Van der Ploeg and Heuvelink, 2005; Schwarz et al., 2010). Sub-optimal temperatures of 8–18°C also negatively affect tomato growth and development due to shorter internodes that restrict plant height, retardation of leaf expansion, reduction of leaf number and total leaf fresh mass and increased dry matter content and thickness of leaves due to increased starch storage (Venema et al., 1999). As reviewed by Schwarz et al. (2010), sub-optimal temperature induces changes in root phytohormone production. This has an impact on root-to-shoot hormone signaling resulting in reduced plant productivity. In addition, temperatures below optimum impair cell membrane fluidity and increase permeability, resulting in ion leakage (Abbas, 2012), while intra- and extracellular water and nutrient movement are inhibited (Mahajan and Tuteja, 2005), reactive oxygen species (ROS) are generated (Gill and Tuteja, 2010), photosynthesis may be restricted (Theocharis et al., 2012), and finally yield is reduced.

Different cultivated tomato varieties may exhibit significant differences in their responses to sub-optimal temperature (Venema et al., 2008). An increase in the tolerance of tomato plants to sub-optimal temperature could considerably reduce the energy cost for growth in heated greenhouses (Venema et al., 2008). Although traditional breeding over the last 30 years resulted in cultivars with twofold improved energy efficiency as a result of increased yield, there is now also a need to reduce the absolute amount of energy input (Van der Ploeg and Heuvelink, 2005). A further successful cultivation of tomato in the field and in unheated greenhouses under lower temperatures requires either the breeding of new cultivars that are better adapted to low temperature or an increase in the tolerance of tomato to sub-optimal temperature aimed to extend the growing period. However, due to low genetic diversity within the cultivated tomato species *Solanum lycopersicum* L. and reduced pollen fertility in interspecific tomato hybrids (Domínguez et al., 2005; Venema et al., 2005), breeding of high-yielding tomato cultivars with enhanced tolerance to sub-optimal temperature has not been successful so far (Schwarz et al., 2010).

An alternative strategy to enhance the tolerance of elite tomato hybrids to sub-optimal temperature is to graft them onto rootstocks that are compatible with the cultivated species and tolerant to sub-optimal temperature. Such rootstocks might be interspecific hybrids of *S. lycopersicum* with accessions of cold-tolerant wild tomato species, such as *S. habrochaites* S. Knapp & D.M. Spooner. The latter species is of particular interest as a potential source of germplasm to widen the genetic variation for low temperature tolerance of cultivated tomato (Bloom et al., 2004; Venema et al., 2005, 2008; Ntatsi et al., 2014a). It originates from an altitude of about 3.200 m (Rick et al., 1994) where an adaptation to low temperatures can be expected (Patterson et al., 1978). The superiority of this cultivar is due to the presence of adaptive mechanisms to alleviate cell damage and to reproduce under sub-optimal temperature.

Comparative analyses on many physiological responses to cold stress were conducted during the last decades proposing several mechanisms that could explain the chilling tolerance of *S. habrochaites* (Venema et al., 2005). Several genetic studies have elucidated the key molecular and physiological mechanisms of cold tolerance in *S. habrochaites* (Goodstal et al., 2005; Arms et al., 2015; Chen et al., 2015). They led to the identification of important QTL controlling shoot wilting under root chilling and the plastochron index, as reviewed by Schwarz et al. (2010). Low temperature has no adverse impact on root growth in high-altitude accessions of wild relatives of *S. lycopersicum* (Venema et al., 2008; Ntatsi et al., 2014a). Grafted plants with the accession LA 1777 of the wild tomato species *S. habrochaites* as the rootstock showed better vegetative growth after exposure to sub-optimal temperatures than plants with *S. lycopersicum* ‘Moneymaker’ as rootstock. Taking this into consideration, the wild species *S. habrochaites* can be proposed as a potential germplasm resource of sub-optimal temperature tolerance in tomato breeding (Venema et al., 1999, 2005, 2008; Foolad and Lin, 2001).

The performance of a grafted plant under sub-optimal temperature conditions is specific for each rootstock/scion combination, because low-temperature tolerance is a complex secondary trait depending on many primary traits (e.g., root and leaf morphology, plant hormones, ROS scavenging compounds, etc.) operating in both the roots and shoots. To elucidate the crucial physiological and molecular mechanisms that determine the positive impact of LA 1777 rootstock on tomato scion performance at sub-optimal temperature, a comprehensive transcriptome analysis is required. Transcriptome profiling in model species such as Arabidopsis has revealed several low temperature stress-related pathways (Chinnusamy et al., 2007; Zhou et al., 2011). In tomato, transcriptome analysis has been used to compare patterns of gene expression under salt, cold, or drought stress (Gong et al., 2010; Sun et al., 2010; Liu et al., 2012). However, to the best of our knowledge, comparative transcriptome analysis of cold-tolerant and -sensitive tomato genotypes under sub-optimal temperature is yet to be reported.

Therefore, we performed a comparative gene expression profiling experiment with the tolerant wild tomato *S*. *habrochaites* accession LA 1777 and the sensitive *S. lycopersicum* cv. Moneymaker as rootstocks and the commercial *S. lycopersicum* cv. Kommeet as scion under optimal and sub-optimal temperatures in the root environment. Our results provide new insights into the molecular mechanisms underlying the sub-optimal temperature tolerance of the wild tomato *S. habrochaites*. This knowledge can be utilized to establish biomarkers to screen not only wild tomato genotypes serving as rootstocks but also rootstock/scion combinations that are likely to be tolerant to sub-optimal temperature (Schwarz et al., 2010).

## Materials and Methods

### Plant Material and Cultivation Conditions

In a glasshouse at the Leibniz Institute of Vegetable and Ornamental Crops, Großbeeren, Germany (latitude 52° 20′ N, longitude 13° 18′ E, altitude 40 m) the commercial tomato cv. Kommeet (KO; De Ruiters, Bergschenhoek, Netherlands) was grafted onto tomato cv. Moneymaker (MM) or onto line accession LA 1777 (LA; C.M. Rick Tomato Genetics Resource Center, UC Davis, CA, United States) of the wild tomato species *S*. *habrochaites*. The grafting resulted in the rootstock/scion combinations MM/KO or LA/KO. Seeds of these three tomato genotypes were germinated in vermiculite for about 20 days. Splice grafting (Savvas et al., 2012) was performed when seedlings had developed 3–4 true leaves. Seventeen days later grafted tomato plantlets were transferred into gullies (8 m × 0.2 m × 0.07 m) in which a standard nutrient solution for tomato (De Kreij et al., 1997) was re-circulating. Prior to transplanting, the roots of the young plantlets were carefully washed in tap water to remove aggregates of the growing medium. The gullies were continuously supplied with nutrient solution which was pumped from a 150 l supply tank at a flow rate of 2 l min^-1^. Twelve plantlets were accommodated in each gully with a density of approximately 2 plants m^-2^. The nutrient solution was replenished on a daily basis. The pH in the re-circulating nutrient solutions was adjusted daily to 5.6–5.7 by adding proper amounts of 1 N HNO_3_ stock solution. The experimental installation started 7 days after planting and was continuously maintained until harvest. Two groups of channels have been used differing in temperature of the re-circulating nutrient solution and thus determining the temperature in the root environment, which was either optimal (day and night 25 ± 0.6°C) or sub-optimal (day and night 15 ± 0.4°C). The target solution temperature of the sub-optimal temperature treatment was accurately maintained by cooling pipes which were connected to the respective solution tanks. The solution temperature of the optimal temperature treatment was determined by the air temperature of the greenhouse. Its daily mean was the same for both treatments at 25 ± 0.8°C with a maximum and minimum at 22.4 and 25.2°C, respectively. Mean relative humidity was 70%, CO_2_ concentration 400 μmol mol^-1^ and mean daily photosynthetically active radiation 12 mol m^-2^ d^-1^.

### Growth Measurements

After 22 days of cultivation at two root temperatures (46 days after grafting), leaf width, shoot length, and number of leaves were measured for two plants in each replication. Total leaf area (*Alp*, m^2^ plant^-1^) was calculated using leaf width of single leaves (Schwarz and Kläring, 2001). Thereafter, harvested plants were divided into stem, leaves and roots (*F*_rp_, g plant^-1^), which were fresh weighed and dried separately at 70°C to a constant mass to measure dry masses. Root samples of about 1 g fresh mass were taken to measure the specific root length (*L*_rw_, m g^-1^) and their mean diameter (*2R*_r_, mm) using an image analyses system (WinRhizo, Regents Instruments, Quebec, Canada). Total root length (*L*_rp_ = *L*_rw_ × *F*_rp_, m plant^-1^) and surface area (*A*_rp_ = *L*_rp_ × π × *2R*_r_, m^2^ plant^-1^) and the specific root area related to fresh mass were calculated (*A*_rw_ = *A*_rp_/*F*_rp_, m^2^ g^-1^).

### Gas Exchange and Chlorophyll Fluorescence Measurements

One day before termination of the experiment, four plants from each grafting treatment were used for leaf gas exchange measurements. The most-recently fully expanded leaf was used in an open gas exchange system (Li-6400, Li-Cor, Inc., Lincoln, NE, United States). Net CO_2_ assimilation (*A*, μmol m^-2^ s^-1^), stomatal conductance (*g*_s_, mmol m^-2^ s^-1^), intercellular CO_2_ concentration (ci), and transpiration rate (*E*, mmol m^-2^ s^-1^) were determined between 9 and 12 a.m., 2 h after the light period started. In the leaf chamber photosynthetic photon flux density was maintained at 1000 μmol m^-2^ s^-1^, relative humidity at 70%, and leaf temperature at 28 ± 0.5°C. Water use efficiency (μmol mmol^-1^) was calculated as *A*/*E*. Chlorophyll fluorescence was measured on the same leaves used for gas exchange measurements after light- or dark-adaptation by employing a pulse amplitude modulated leaf chamber fluorometer (Li-6400). Minimal fluorescence values in the dark-adapted state (*F*_o_) were obtained by application of a low-intensity red measuring light source (630 nm), whereas maximal fluorescence values (*F*_m_) were measured after applying a saturating light pulse of 8.000 μmol m^-2^ s^-1^, and thus maximum quantum use efficiency of PSII in the dark-adapted state was calculated as *F*_v_ = *F*_m_ -*F*_o_. The leaf area assayed was dark-adapted for at least 30 min prior to *F*_v_/*F*_m_ measurements. Minimum (*F*′_o_) and maximum (*F*′_m_) values of fluorescence in the light-adapted state at 800 μmol m^-2^ s^-1^ were also obtained. After leaves were continuously illuminated with actinic light for 6 min, the steady-state fluorescence (*F*_s_) was recorded. Using these parameters, the following ratios were calculated: effective quantum use efficiency of PSII in the light-adapted state as F_v_′/F_m_′ = (F_m_′- F_o_′)/F_m_′, effective quantum yield as Φ_PSII_ = (F_m_′- F_s_)/F_m_′ photochemical quenching as *q*_P_ = (F_m_′- F_s_)/(F_m_′- F_o_′), and non-photochemical quenching as NPQ = (F_m_ - F_m_′)/F_m_′.

### Analyses of Carbohydrates and Characteristics of Oxidative Stress

Soluble sugars were measured according to Geigenberger and Stitt (1993), starch according to Sonnewald (1992), and total amino acids according to Moore and Stein (1948) using a Synergy HT 96-position microplate spectrophotometer (BioTek instruments GmbH, Winooski, VT, United States).

Hydrogen peroxide (H_2_O_2_) was determined according to the method described by Mukherjee and Choudhuri (1983) and electrolyte leakage as described by Lutts et al. (1995). Lipid peroxidation was determined in terms of concentration of thiobarbituric acid-reactive substances and quantified on its product, malondialdehye according to the method described by Hodges et al. (1999) and He et al. (2009).

For enzyme analysis, 0.1 g of each pulverized, frozen sample (leaf or root) was homogenized with ice-cold 25 mM HEPES buffer (pH 7.8) including 0.2 mM EDTA, 2 mM ascorbate and 2% (w/v) polyvinylpyrrolidon (PVP). The homogenate was centrifuged at 4°C and 14.000 rpm for 5 min. The supernatants were used for enzyme analysis. All steps in the preparation of enzyme extract were carried out at 4°C. Protein content was determined as described by Bradford (1976) with bovine serum albumin as standard. Ascorbate peroxidase activity was determined according to Nakano and Asada (1981) as decrease in absorbance at 290 nm due to ascorbate oxidation (*E* = 2.8 mM^-1^ cm^-1^) and the reaction was initiated by adding H_2_O_2_. Catalase activity was measured according to Cakmak and Marshner (1992), as modified by Ogweno et al. (2009). Briefly, 200 μl of reaction mixture containing 25 mM potassium phosphate buffer (pH 7.0), 0.1 mM EDTA, 10 mM H_2_O_2_ and 10 μl plant extract were used. The decline in absorbance at 240 nm due to the decomposition of H_2_O_2_ was measured for 5 min (*E* = 39.4 mM^-1^ cm^-1^). Guaiacol peroxidase (G-POD) activity was determined according to Cakmak and Marshner (1992) as modified by Egley et al. (1983). Increase in absorbance at 510 nm caused by guaiacol oxidation (*E* = 26.6 mM^-1^ cm^-1^) was measured over 50 min. Glutathione reductase (GR) activity was assayed according to Rao et al. (1996), with some modifications. Briefly, the reaction mixture in a total volume of 200 μl consisted of 25 mM potassium buffer (pH 7.0), 0.1 mM EDTA, 0.5 mM oxidized glutathione, 0.12 mM NADPH and 10 μl plant sample. GR activity was measured by following the decrease in absorbance of oxidized glutathione at 340 nm (*E* = 6.2 mM^-1^ cm^-1^). Superoxide dismutase (SOD) activity was determined using the method of Rao and Sresty (2000). One unit of enzyme activity was defined as the amount of enzyme required to result in a 50% inhibition in the rate of nitro blue tetrazolium reduction measured at 560 nm.

### Microarray Analysis

For microarray analysis leaf and root samples from all treatments were collected 20 days after employing temperature treatments between 10 and 11 a.m. and immediately frozen in liquid nitrogen and stored at -80°C until use. Twelve independent biological samples for each treatment were harvested from leaves (third and fourth) and roots, and then four samples were pooled to obtain three replicates per treatment and organ for total RNA isolation. Leaf and root material was homogenized using a ball mill (Retsch, Haan, Germany) and RNA was extracted with the RNAeasy Plant Mini Kit (Qiagen GmbH, Hilden, Germany). RNA concentration and quality were determined using a spectrophotometer (Nanodrop Technologies, Wilmington, DE, United States) and a bioanalyzer (Agilent, Santa Clara, CA, United States), respectively. cRNA synthesis, labeling, hybridization onto Agilent 4x44k 022270 tomato microarrays^1^ and array scanning were performed at ATLAS Biolabs GmbH (Berlin, Germany). A total of 24 samples were used for the hybridizations.

The raw microarray data were processed using the Robin application (Lohse et al., 2010) to check hybridization quality and identify differentially expressed genes (DEG). Four samples were excluded from downstream analyses because they showed strong outlier behavior in the quality checks. Statistical assessment of differential gene expression was performed using default settings in Robin. Briefly, raw data were normalized by applying the Robust Multichip Average method (Irizarry et al., 2003), and subsequently, DEG were identified using the limma R package (Smyth, 2004). Genes showing an absolute log2 fold change greater than 1 and a false discovery rate corrected *p*-value (Benjamini and Hochberg, 1995) below 0.05 were accepted as significantly differentially expressed.

Assignment of the different genes represented by identifiers to respective bins and visualization of data sets was realized using MapMan software (Thimm et al., 2004). Significantly changed genes were divided into up-regulated and down-regulated genes and differences in gene expression were visualized using MapMan (Thimm et al., 2004; Usadel et al., 2005). The averaged log signal values of all treatments are presented as heatmaps generated by Microsoft Office Excel Software 2007. Enrichment of DEG in functional categories of MapMan bins was tested using the Mefisto software including Bonferroni correction for multiple testing. The probe sequences of DEG were retrieved manually using the NCBI GenBank accession numbers from the Agilent source IDs. To determine the Unigene Identification and gi numbers BLAST searches were performed using sequences as query obtained from NCBI against SGN tomato DB contained in the SGN whole genome database^2^. Microarray hybridization results are available at ArrayExpress^3^ under accession number E-MTAB-4986.

### Further Statistical Analyses

For all other data the two temperature treatments were combined with the two grafting combinations in a two-factorial experimental design with four replications per treatment and three plants per replication. Initially, data were subjected to factorial analysis of variance. When the temperature in the root environment and/or the grafting combination had a significant impact but the interaction between them was insignificant, the means between the two tested temperatures and/or the two grafting combinations were separated using the Duncan’s Multiple Range Test (*p* < 0.05). The same test was used to separate means of all four treatments when also the interaction was significant. All statistical analyses were carried out using the STATISTICA software package, version 9.0.

## Results

### Differences in Growth and Physiological Traits between Grafting Combinations Using Sub-Optimal Temperature Tolerant and Sensitive Tomato Rootstock Genotypes

Both sub-optimal temperature and grafting onto LA 1777 reduced shoot fresh and dry mass and total leaf area of the commercial tomato cultivar Kommeet (**Table 1**). Temperature interacted with the grafting combination on root traits. In particular under optimal temperature the use of ‘Moneymaker’ as rootstock was associated with significantly higher levels of root fresh and dry mass in comparison to LA 1777. Under sub-optimal temperature these differences between the two grafting combinations were no longer observed (**Table 1**) because root fresh and dry mass were increased 2.2 and 1.6-fold at sub-optimal temperature in LA/KO, while they were 2.0 and 1.5-fold decreased in MM/KO relative to the optimal temperature condition. Consequently, the root/shoot ratio was significantly higher (doubled) for LA/KO compared to MM/KO at sub-optimal temperature while it was the same at optimal temperature. Other root characteristics (Supplementary Table 1) were not influenced either by the temperature treatment or by the rootstock, except for average root diameter, which was highest under sub-optimal temperature regardless of the rootstock.

**Table 1:** Effects of exposure of tomato cultivar Kommeet grafted onto LA 1777 (LA/KO) or ‘Moneymaker’ (MM/KO) to sub-optimal and optimal temperature (T) in the root environment on fresh (FM), dry mass (DM), and dry matter content (DMC) of shoot and root, total leaf (*A*_lp_) and root surface area (*A*_rp_).

Treatment		Shoot				Roots			
		FM, g	DM, g	DMC, %	*A*_lp_, m^2^	FM, g	DM, g	DMC, %	*A*_rp_, m^2^
Sub-optimal T	LA/KO	63.42	4.77	7.52ˆa	1.56ˆb	16.08ˆb	0.65ˆb	4.06ˆc	0.363ˆb
	MM/KO	126.32	8.38	6.64ˆb	2.58ˆb	14.13ˆb	0.73ˆb	5.14ˆb	0.334ˆb
Optimal T	LA/KO	79.45	5.23	6.58ˆb	1.79ˆb	7.11ˆc	0.40ˆc	5.60ˆa	0.165ˆc
	MM/KO	212.73	14.03	6.60ˆb	4.89ˆa	28.32ˆa	1.26ˆa	4.43ˆbc	0.423ˆa
**Main effects**								
T	Sub-optimal	94.87	6.58	6.93	2.14	15.10	0.69	4.66	0.348
	Optimal	146.09	9.63	6.59	3.12	17.72	0.83	5.92	0.294
R/S	LA/KO	71.43	5.00	6.99	1.69	11.60	0.53	5.01	0.264
	MM/KO	169.53	11.21	6.61	3.57	21.22	0.99	5.57	0.378
**Statistical significance**								
T		ˆ*	^∗^	^∗^	^∗^	ns	ns	^∗^	^∗^
R/S		ˆ**	^∗∗^	^∗^	^∗∗∗^	^∗^	^∗^	^∗^	^∗^
T × R/S		ns	ns	^∗^	^∗^	^∗^	^∗^	^∗^	^∗∗^

*Grafted plants were obtained using ‘Moneymaker’ (MM) or LA 1777 (LA) as rootstock and ‘Kommeet’ (KO) as scion in the rootstock/scion combinations (R/S). Different letters within the same column indicate significant differences between means of four replicates according to the Duncan’s Multiple Range Test (*p* ≤ 0.05). ns, ^∗, ∗∗^, and ^∗∗∗^ indicate non-significant or significant differences at *P* ≤ 0.05, *P* ≤ 0.01, and *P* ≤ 0.001, respectively.*

The rates of net CO_2_ assimilation and transpiration of the leaves did not respond to sub-optimal root temperature, regardless of the rootstock (Supplementary Table 2). Moreover, chlorophyll fluorescence measurements of light-adapted and dark-adapted leaves (Supplementary Table 3) and soluble carbohydrate content (Supplementary Table 4) were not influenced either by the temperature or by the rootstock. A significant increase in stomatal conductance (*g*_s_) occurred when roots were subjected to sub-optimal temperature, while *g*_s_ of LA/KO was significantly reduced compared with MM/KO. Water use efficiency was significantly increased and intracellular CO_2_ was significantly reduced at sub-optimal root temperature, regardless of the rootstock.

The activity of G-POD in tomato roots was elevated when the rootstocks were subjected to sub-optimal temperature. The activities of G-POD in leaves and GR and SOD in leaves and roots of tomato were not influenced by either the temperature or the rootstock. However, in the roots of LA 1777 the increase of G-POD at sub-optimal compared with optimal temperature was 75% and thus, much higher than in ‘Moneymaker,’ were it was only 24% (Supplementary Table 5). Total amino acids, electrolyte leakage and starch content were not influenced in leaves and roots either by the temperature or by the rootstock (Supplementary Table 6). The same pattern was also found for lipid peroxidation, H_2_O_2_ and total protein content in leaves and roots (Supplementary Table 7).

### Differences in Global Gene Expression between Sub-Optimal Temperature Tolerant and Sensitive Tomato Genotypes

**FIGURE 1:**
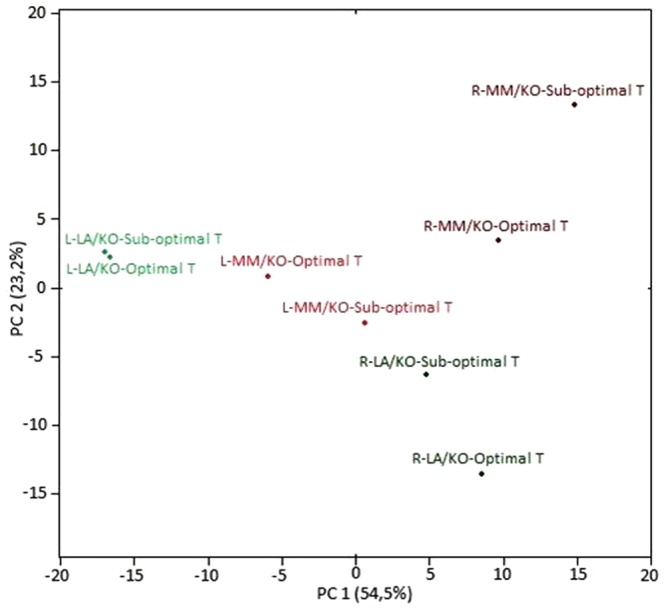
Principal component (PC) analysis of transcript profiles of leaves (L) and roots (R) of ‘Kommeet’ grafted onto LA 1777 (LA/KO) or onto ‘Moneymaker’ (MM/KO) following treatments of grafting combination (R/S) and temperature in the root environment (T).

Principal component analysis (PCA) was used to identify patterns of global gene expression in leaves and roots of grafted plants (**Figure 1**). PC1 clearly separated the scores of normalized transcript data for leaves from those for root samples and explained 54.5% of the total variance. PC2 explained 23.2% of the variance and additionally separated the rootstocks from each other and also the different temperature treatments. Interestingly, under sub-optimal temperature the transcript profile of leaves of ‘Kommeet’ grafted onto LA 1777 (LA/KO) was clearly separated from the profile of ‘Kommeet’ plants grafted onto ‘Moneymaker’ (MM/KO), indicating transcriptional differences in the leaves of the two grafting combinations when the roots were exposed to low temperature. Moreover, the transcript profile of LA/KO leaves grown under optimal temperature clustered closely to that of leaves grown under sub-optimal temperature (**Figure 1**). The distance between scores for LA/KO or MM/KO roots subjected either to optimal or sub-optimal temperature was larger compared to leaf samples of the same grafting combination indicating strong transcriptional changes induced in roots after a temperature shift of 10°C.

**FIGURE 2:**
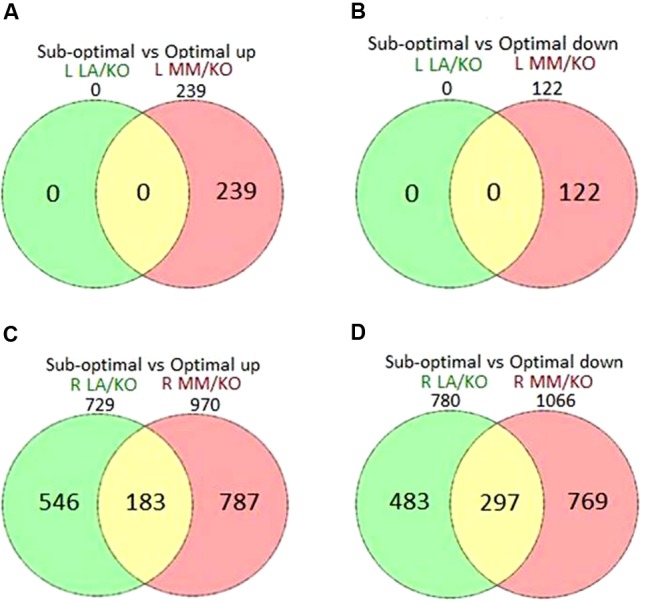
Venn diagrams showing differentially up-regulated **(A,C)** or down-regulated genes **(B,D)** in leaves (L) **(A,B)** or roots (R) **(C,D)** of ‘Kommeet’ grafted onto LA 1777 (LA/KO) or ‘Moneymaker’ (MM/KO) at sub-optimal temperature in the root environment. The numbers above the circles indicate the total number of up- or down-regulated genes at sub-optimal temperature in each grafting combination.

Significantly (FDR *p* < 0.05, log2 fold change above 1 or below -1) DEG in leaves and roots of the grafting combinations at sub-optimal compared to optimal temperature are shown in Venn diagrams (**Figure 2**). After 22 days of sub-optimal temperature in the root environment, a total of 361 (239 up- and 122 down-regulated) genes were significantly differentially expressed in the leaves of MM/KO, while in the leaves of LA/KO no significantly DEG were identified (**Figures 2A,B**) in agreement with the PCA results (**Figure 1**). In the roots of MM/KO a total of 2036 significantly regulated genes (970 up and 1066 down) were identified, while only 1509 (729 up- and 780 down-regulated) cold responsive genes were observed in the roots of LA/KO (**Figures 2C,D**). Among them, 1029 cold-responsive genes (546 up- and 483 down-regulated) were exclusively identified in the roots of LA/KO, whereas 1556 genes (787 up- and 769 down-regulated) were uniquely observed in roots of MM/KO. A total of 480 genes (183 up- and 297 down-regulated) were commonly regulated at sub-optimal temperature in both tomato grafts (**Figure 2**).

### Identification of Sub-Optimal Temperature Responsive Genes in Leaves and Roots of the Graft Combinations LA/KO or MM/KO

**FIGURE 3:**
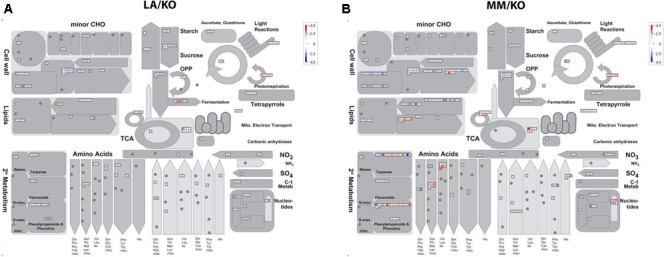
Assignment of differentially expressed genes upon different temperatures in the roots of the grafting combination ‘Kommeet’ grafted onto LA 1777 (LA/KO) **(A)** and onto ‘Moneymaker’ (MM/KO) **(B)** to the functional category metabolism using MapMan. Log2-fold expression differences for individual genes are plotted into bins grouped according to their putative functional annotation. Squares within bins are colored red for an up-regulated and blue for a down-regulated gene with darker color reflecting a stronger differential expression.

**FIGURE 4:**
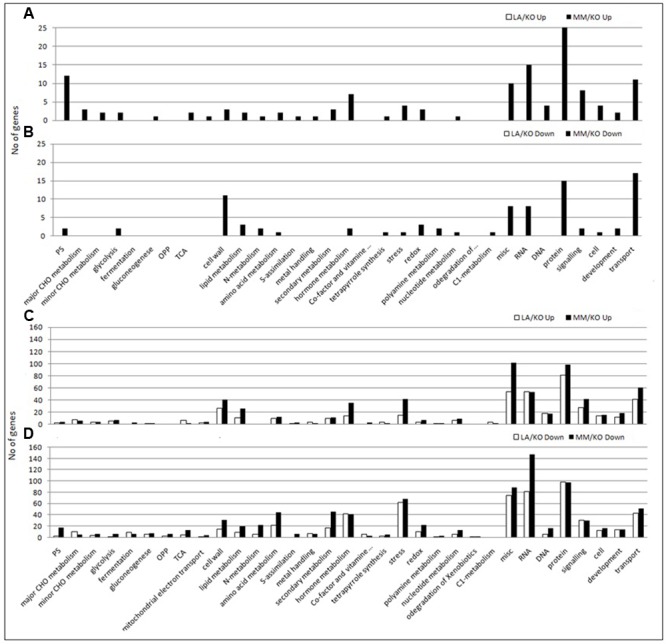
Number of up- **(A,C)** or down-regulated **(B,D)** genes (log2 ratio sub-/optimal temperature ≥1 or ≤-1 and *p*-value < 0.05) in MapMan functional categories. Shown is the number of genes in leaves **(A,B)** and roots **(C,D)** of the graft combination LA 1777/‘Kommeet’ (LA/KO) and ‘Moneymaker’/‘Kommeet’ (MM/KO). Only genes with known function could be assigned to a MapMan category and were used.

Genes up- or down-regulated in response to low temperature in leaves and roots were assigned to functional categories using the MapMan software. As an example, the assignment to the functional category metabolism of up- and down-regulated genes in roots of either LA 1777 or ‘Moneymaker’ is presented in **Figure 3**. After 22 days of sub-optimal temperature in the root environment, DEG were only detected in leaves of MM/KO (**Figure 2A**). However, from the 239 genes up-regulated in MM/KO more than half (131) could not be linked to any category since they represent genes of unknown function. All other genes could be assigned to the following bins: protein synthesis, degradation and post-translational modification (25 genes); RNA regulation of transcription (15 genes); photosynthesis light reaction photosystem I, and Calvin cycle (12 genes), transport (11 genes), signaling (8 genes), hormone metabolism (7 genes), and miscellaneous (10 genes) (**Figure 4A** and Supplementary Table 8). All other categories were present with two to four up-regulated genes. Of the 122 down-regulated genes in leaves of MM/KO, 85 were unknown. The other gene products were assigned to the bins transport (17 genes), protein synthesis, degradation and post-translational modification (15 genes), cell wall (11 genes), RNA regulation of transcription and miscellaneous (8 genes each) (**Figure 4B** and Supplementary Table 9).

In the roots of both graft combinations more up-regulated genes were identified compared to leaves, with 729 for the combination LA/KO with 299 genes with unknown function and 970 for MM/KO including 353 genes with unknown function. The largest proportion of genes with known function belonged to the category protein synthesis, degradation and post-translational modification (81 out of 430 genes in LA/KO and 98 out of 627 in MM/KO), followed by RNA regulation of transcription (54 or 53 genes) and transport (41 or 60 genes) (**Figure 4C** and Supplementary Tables 10, 12). The functional categories signaling (27 or 41 genes), cell wall (26 or 40 genes), stress (15 or 41 genes), were also highly represented.

One hundred and eighty-four out of 780 down-regulated genes in the roots of the graft combination LA/KO and 211 out of 1056 down-regulated genes in the combination MM/KO subjected to sub-optimal root temperature were unknown. Most of the other down-regulated genes were assigned to the most abundant category protein synthesis, degradation and post-translational modification (98 genes each out of 596 in LA/KO and 98 out of 845 in MM/KO), followed by RNA regulation of transcription (81 or 148 genes), stress (62 or 68 genes), transport (43 or 51 genes), hormone metabolism (42 or 41 genes), and signaling (27 or 29 genes) (**Figure 4D** and Supplementary Tables 11, 13).

### Enrichment of Sub-Optimal Temperature Responsive Genes of Roots in MapMan Functional Categories

Enrichment of up-regulated genes in MapMan functional categories differed widely between the roots of the two graft combinations (**Table 2**). Only two categories were commonly over-represented in both genotypes, cell wall and unassigned genes. Whereas in the combination LA/KO only two additional sub-bins of the cell wall bin were over-represented, in the combination MM/KO eight further over-represented bins were found, including lipid metabolism, fatty acid desaturation and hormone metabolism, biotic stress, miscellaneous genes from the functional category cytochrome P450 as well as protein degradation of AAA proteins.

**Table 2:** Over-representation analysis (Mefisto) of significant differentially expressed genes in roots subjected to sub-optimal root temperature in the two graft combinations LA 1777/’Kommeet’ (LA/KO) and ‘Moneymaker’/‘Kommeet’ (MM/KO) visualized in MapMan functional categories (to sub-category 3).

Bin code	Bin name	Up-regulated		Down-regulated	
		LA/KO	MM/KO	LA/KO	MM/KO
10	Cell wall	0.005	<0.001	ns	ns
10.2	Cell wall.cellulose synthesis.	<0.001	ns	ns	ns
10.2.1	Cell wall.cellulose synthesis.cellulose synthase	0.009	ns	ns	ns
11.2	Lipid metabolism.FA desaturation	ns	0.003	ns	ns
16	Secondary metabolism	ns	ns	<0.001	0.011
16.1.5	Secondary metabolism.isoprenoids.terpenoids	ns	ns	0.009	0.009
16.2	Secondary metabolism.phenylpropanoids	ns	ns	<0.001	<0.001
17	Hormone metabolism	ns	0.018	<0.001	0.004
17.4	Hormone metabolism.cytokinin	ns	ns	<0.001	<0.001
17.4.2	Hormone metabolism.cytokinin.signal transduction	ns	ns	0.005	0.002
17.5	Hormone metabolism.ethylene	ns	ns	0.002	<0.001
17.5.1	Hormone metabolism.ethylene.synthesis-degradation	ns	ns	<0.001	0.021
20	Stress	ns	0.044	<0.001	0.002
20.1	Stress biotic	ns	<0.001	ns	<0.001
20.2	Stress.abiotic	ns	ns	<0.001	<0.001
20.2.1	Stress.abiotic.heat	ns	ns	<0.001	<0.001
26	Misc	ns	<0.001	ns	ns
26.1	Misc.cytochrome P450	ns	0.011	ns	ns
27	RNA	ns	ns	0.001	<0.001
27.3	RNA.regulation of transcription	ns	ns	<0.001	0.004
27.3.26	RNA.regulation of transcription. MYB-related transcription factor family	ns	ns	0.018	ns
29	protein	ns	ns	<0.001	<0.001
29.2	Protein.synthesis	ns	ns	0.002	<0.001
29.5.9	Protein.degradation.AAA type	ns	0.001	ns	ns
35	Not assigned	ns	<0.001	ns	ns
35.2	Not assigned.unknown	0.01	<0.001	0.049	0.034

*Significantly over-represented functional bins are given with their adjusted *p*-values (Bonferroni correction). ns, not significant.*

For down-regulated genes the overlap between the differently tolerant rootstocks was much higher, with 16 common over-represented categories. Here the MapMan functional categories that were significantly enriched in both rootstock genotypes were secondary metabolism especially of isoprenoids (particularly terpenoids) and phenylpropanoids, hormone metabolism associated with cytokinin and cytokinin signal transduction as well as with ethylene synthesis and degradation, biotic and abiotic stress response, RNA regulation of transcription and protein synthesis. In addition RNA regulation of transcription of MYB domain transcription factor family is particularly enriched in the down-regulated genes of the LA 1777 rootstock together with not assigned genes, while genes associated with biotic stress were enriched only in the down-regulated genes of ‘Moneymaker’ roots subjected to sub-optimal temperature (**Table 2**).

### Genes Differentially Expressed in Response to Sub-Optimal Temperature Differ between the Tolerant and Sensitive Rootstock Genotype

Among the 480 genes regulated by sub-optimal temperature in roots only 13 were highly differentially expressed between the roots of the tolerant and the sensitive rootstock (difference of log2 fold change ≥|2|) (**Table 3** and Supplementary Table 14). These genes were from a variety of functional groups encoding a salicylic acid carboxyl methyltransferase (*SGN-U572374*), an F-box protein (*SGN-U578297*), a CYP72A15 cytochrome P450 (*SGN-U580908*), and a sigma factor binding protein 1 (*SGN-U565390*), all strongly increased in expression in MM/KO roots but decreased in LA/KO roots in response to low temperature. Several other genes showed a weaker differential regulation between the genotypes. However, genes encoding a putrescine *N*-methyltransferase (*SGN-U566249*) and a CYP71A22 cytochrome P450 (*SGN-U575254*) were more strongly down-regulated in MM/KO than in LA/KO under sub-optimal temperature. In addition the gene encoding an ethylene-responsive heat shock protein cognate 70 (*SGN-U579872*) was reduced in both genotypes but much stronger in the tolerant rootstock LA 1777.

**Table 3:** Differentially expressed genes between optimal and sub-optimal temperature in LA 1777 (LA) and ‘Moneymaker’ (MM) rootstocks (*p* < 0.05, difference log2 fold change > | 1|).

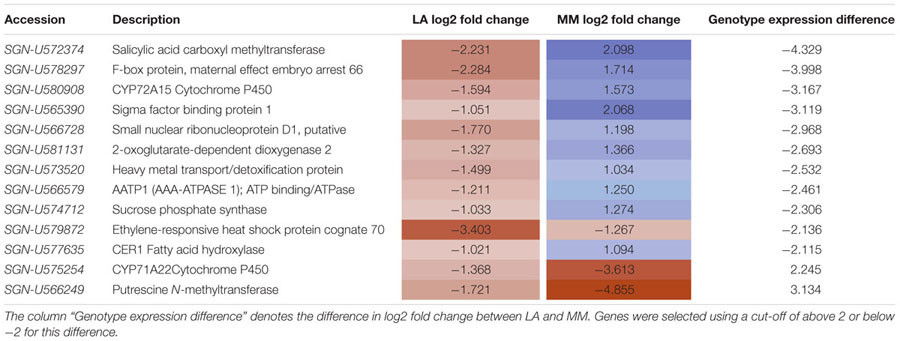

## Discussion

In this study, transcript profiling and analysis of growth, and physiological responses of a commercial tomato cv. Kommeet grafted onto two rootstocks differing in their tolerance to sub-optimal temperature were combined to achieve a more comprehensive understanding of the long-term stress responses of tomato rootstocks and scions under sub-optimal temperature. The present findings complement and extend previous research, where the positive impact of the high-altitude accession LA 1777 (*S. habrochaites*) used as a rootstock on tomato scion performance at sub-optimal temperature has been demonstrated but was limited to plant growth and selected physiological traits (Venema et al., 2005, 2008; Ntatsi et al., 2014a,b).

### Grafting on Differently Sub-Optimal Temperature Tolerant Rootstocks Causes Differences in Growth and Physiological Traits

The exposure of tomato grafts to sub-optimal root temperature resulted in a profound decrease of shoot fresh and dry mass in agreement with previous studies (Venema et al., 2008; Ntatsi et al., 2013). The supply of growth supporting assimilates was not limited by the exposure of the plants to sub-optimal temperature, as the rates of net CO_2_ assimilation in leaves and the starch content in leaves and roots were not decreased by the stress applied in the root environment. Hence, the present study is in agreement with the view that other factors than the production and availability of assimilates restrict shoot growth under sub-optimal temperature (Ntatsi et al., 2013).

As already reported by Venema et al. (2008) grafting tomato scions onto LA 1777 improved shoot growth at sub-optimal temperature. Low temperature also affects root growth, size, and architecture (Nagel et al., 2009). Changes in root morphology were interpreted as adaptation of nutrient acquisition mechanisms to low temperature, aiming at extending the absorbing surface area per unit root mass or length (Macduff et al., 1986). Thus, formation of a more extensive root system by tomato plants grafted onto LA 1777 in the cold, and the concomitant increase in root/shoot ratio, provides an advantage in terms of nutrient and water uptake. In the present study sub-optimal root temperature resulted in an approximately 2.8-fold increase of the root/shoot ratio when plants were grafted onto LA 1777 while no increase was observed when ‘Moneymaker’ was used as the rootstock. In contrast, in previous studies no impact of sub-optimal temperature on root growth of LA 1777 was observed (Bloom et al., 1998, 2004).

An increase in root/shoot ratio has been interpreted by Equiza et al. (2001) as a mechanism to overcome restrictions in water absorption. Under sub-optimal temperatures an imbalance between root water uptake and leaf transpiration may occur. According to Aroca et al. (2001) chilling sensitivity differences among maize genotypes can be ascribed to different responses of the root water uptake rate. Indeed, when the temperature falls below the optimum, root water uptake decreases due to a decrease of vapor pressure difference (Aroca et al., 2003), while water viscosity increases (Bloom et al., 2004). According to the same authors, although transpiration decreases, the stomata of the sensitive maize and tomato genotypes remain open, while those of tolerant genotypes close rapidly, indicating an adaptive mechanism to the decline in water movement caused by sub-optimal temperature. In this work the stomatal conductance of ‘Kommeet’ scions was clearly affected by the rootstock, with lower stomatal conductance when grafted onto the sensitive ‘Moneymaker’ in comparison to the tolerant LA 1777. For grafted plants it was shown that shoots as well as roots contribute to shoot turgor maintenance during root chilling (Bloom et al., 2004). Grafted plants with roots containing *S. habrochaites* and *S. lycopersicoides* Dunal introgressions improved shoot turgor maintenance under root chilling (Easlon et al., 2013).

Under low temperature stress plant tissues accumulate metabolites such as sugars, amino acids, or secondary metabolites like flavonoids (Zhang et al., 2007; Ma et al., 2009; Schulz et al., 2015). In this study shoots or roots did not accumulate sugars or amino acids indicating that the low temperature treatment applied only to the rootstocks was not sufficiently severe or that after 22 days of sub-optimal temperature treatment concentrations of these compounds returned to control values. Another study on leaves of ‘Moneymaker’ subjected to 10°C for up to 48 h also did not observe a significant accumulation of compatible sugars and suggested that the accumulation of amino acids and polyamines might play a relevant role in tomato chilling acclimation (Barrero-Gil et al., 2016).

Also lipid peroxidation and antioxidant enzyme activity were not changed in leaves or roots after 22 days of sub-optimal root temperature. The maximum lipid peroxidation occurs in tomato after 10 days of cold stress accompanied by a gradual increase of SOD activity, suggesting that oxidative stress may only play a key role in short-term responses of tomato to cold (Aydin et al., 2013).

### Sub-Optimal Temperature Caused Expression Changes in Rootstocks of Both Genotypes But Only in Scion Leaves When Grafted onto the Sensitive Rootstock

A PCA of our gene expression data identified discrete responses of scions and rootstocks of grafted plants subjected to sub-optimal root temperature, and a distinct separation between LA 1777 and ‘Moneymaker’ rootstocks. Under sub-optimal root temperature significant transcriptional changes in leaves were only induced in the scion ‘Kommeet’ when grafted onto the sensitive rootstock ‘Moneymaker.’ This clearly demonstrates the impact of the rootstock genotype on the transcriptional responses of the scion even when the low temperature stress is only applied to the roots. Moreover, roots subjected to sub-optimal temperature were clearly separable by PCA from roots grown under optimal temperature in both genotypes, indicating that a temperature shift of 10°C in the root environment imposed comprehensive changes at transcript level. The number of DEG in ‘Moneymaker’ roots was 4.0 or 8.6-fold higher for up- or down-regulated genes, respectively, compared to the number of DEG in leaves, suggesting that the main transcriptional cold response occurred in the cold exposed roots and not in the leaves, which were exposed to optimal conditions. However, also in rice the number of DEGs identified in roots in response to a cold treatment of whole plants was larger than those in shoots in both a tolerant and a sensitive cultivar (Yang et al., 2015), indicating that at least in chilling-sensitive plants roots may respond more strongly to cold than leaves.

The number of DEG in response to sub-optimal root temperatures in leaves of ‘Kommeet’ was much lower compared to 3350 or 2940 increased and 3022 or 3126 decreased transcripts in leaves of *S. lycopersicum* or *S. habrochaites* after 12 h of cold treatment of whole plants at 4°C (Chen et al., 2015). The early transcriptomic response in leaves of 3-week-old ‘Moneymaker’ plants to 3, 24, and 48 h at 10°C revealed 1537, 4404, and 4600 DEGs, respectively, with more up- than down-regulated genes (Barrero-Gil et al., 2016). No studies on the influence of sub-optimal root temperature on roots of tomato are available for a direct comparison.

### Identification of Sub-Optimal Temperature Responsive Genes in Leaves of the Graft Combination MM/KO

Only 10 of the cold up-regulated DEG in leaves of the graft combination MM/KO showed a log2 fold change above two and most of them had an unknown function. Of the down-regulated genes only one had a log2 fold change below two, but here certain groups of genes could be detected in the list of the 20 most highly down-regulated genes. Two genes encoding glutaredoxin family proteins involved in salicylic acid signaling (Rouhier et al., 2008) were detected together with genes encoding JAZ3 and CRF3, two proteins involved in hormone metabolism, and *Xth23* encoding a xyloglucan endotransglycosylase 6 as well as *FAD2* (fatty acid desaturase 2). In general, cold influenced gene expression in leaves of a scion grafted on a sensitive rootstock in the functional categories biotic defense response, hormone and cell wall metabolism.

In contrast, analysis of DEG detected in leaves of ‘Moneymaker’ during an exposure to 10°C for 3 h indicated that transcription factor activity dominated the early response together with GO terms for stress response and hormone biosynthesis and signaling (Barrero-Gil et al., 2016). In addition, metabolism was adjusted by accumulation of compatible solutes, activation of antioxidants systems and rearrangement of the photosynthetic machinery (Barrero-Gil et al., 2016). These early stress responses were not found in the present study, indicating that after a cold treatment of 10°C for 22 days no acute stress responses can be observed anymore and that the plants have probably reached a new homeostasis.

### Enrichment of DEGs in Functional Categories in Roots Reflects Down-Regulation of Main Stress Responsive Functions and Activation of Cellulose Synthesis Related Genes in the Tolerant Rootstock Whereas Sensitive Rootstocks Show Stress Responses

Cellulose constitutes the major part of plant cell walls, determining cell shape and plant morphology and is synthesized by cellulose synthase complexes localized on the plasma membrane (Doblin et al., 2002). Up-regulated genes were significantly enriched in the MapMan functional category of cell wall synthesis in both rootstock genotypes. Enrichment in the category cellulose synthesis and the sub-bin cellulose synthase, however, was only observed for the tolerant rootstock, suggesting that cellulose synthesis may be one of the main adaptation mechanisms to long-term sub-optimal temperature. In tomato leaves cellulose biosynthesis and cell wall organization are the main functions of target genes of miRNAs differentially expressed after short term cold treatment (up to 48 h) (Cao et al., 2014). Also, a gene encoding a cellulose synthase subunit is targeted by a chilling-responsive miRNA in tomato with an essential role in the low temperature response (Cao et al., 2014). Plant growth and development is immediately inhibited under cold stress, resulting in down-regulated cell wall formation. A later up-regulation of the corresponding genes may be necessary for a long-term cold adaptation. This hypothesis is in agreement with the finding that sub-optimal temperature has no adverse impact on root growth of LA 1777 (Venema et al., 2008). Cell wall remodeling was also found in Arabidopsis as a long-term adaptation mechanism to cope with multiple biotic or abiotic stresses (Atkinson et al., 2013). Additionally proteomic analysis of two differently tolerant cotton cultivars identified cellulose synthesis as important for the tolerance of cotton fibers to low temperature (Zheng et al., 2012).

In both grafting combinations down-regulated genes in the rootstocks were overrepresented in bins related to secondary metabolism, especially isoprenoids, terpenoids and phenylpropanoids. This was unexpected since the accumulation of flavonoids and anthocyanins involved in ROS scavenging is an important adjustment during cold acclimation (Ruelland et al., 2009; Schulz et al., 2016). Several genes encoding enzymes involved in flavonol and anthocyanin biosynthesis were up-regulated during cold treatment in tomato leaves together with numerous genes coding for enzymes with antioxidant activity, in agreement with increased anthocyanin levels (Barrero-Gil et al., 2016). Whereas no corresponding data are available for tomato roots, expression of genes encoding enzymes involved in secondary metabolism, especially phenylpropanoid and lignin biosynthesis, is increased in the roots of a cold-tolerant compared to a sensitive rice cultivar after a short-term cold stress (Yang et al., 2015). The apparent down-regulation of secondary metabolism in tomato roots after long-term cold treatment may be an adaptive, time-dependent response. However, this hypothesis needs to be investigated further.

The hypothesis that perceived stress levels were reduced during acclimation to low temperature in the rootstocks of both cultivars was supported by an enrichment of down-regulated genes in the abiotic and biotic stress bins. This effect of long-term stress is further supported by the fact that bins for regulation of transcription and protein synthesis were enriched for down-regulated genes, while they were enriched for up-regulated genes after a short-term cold stress in the roots of a tolerant rice cultivar (Yang et al., 2015).

Rootstocks of the sensitive cultivar Moneymaker showed in addition an enrichment of up-regulated genes in the bins fatty acid desaturation, phenylpropanoids, biotic stress, cytochrome P450 and protein degradation, indicating that the sensitive cultivar needed more transcriptional adaption to low temperature than the tolerant cultivar that did not show these reactions.

Studies analyzing the effects of low temperature on root-derived phytohormones clearly demonstrate a strong impact on both root growth and root-to-shoot signaling, imposing alterations in shoot physiology and thus productivity (Aloni et al., 2010; Schwarz et al., 2010; Ntatsi et al., 2014a). However, due to intensive hormone cross-talk (positive or negative), individual processes are affected by multiple hormones. Under stress conditions the levels of a range of hormones, divided into the groups of “positive growth regulators” (auxin, cytokinins, and brassinosteroids) and “stress hormones” [abscisic acid (ABA), jasmonic acid, salicylic acid and ethylene] are changing. Interestingly, the functional category of cytokinin metabolism was significantly enriched for down-regulated genes in the roots of both genotypes, in agreement with the finding that low temperature resulted in a sharp decline in cytokinin concentration in the shoots of wheat plants due to increased activity of cytokinin oxidase (Veselova et al., 2005). However, in the present study down-regulated genes were mainly overrepresented in the functional category of cytokinin signal transduction. A multistep two-component signaling system functions as a key element of cytokinin signaling in Arabidopsis in the early cold stress response (Jeon et al., 2010). Since we analyzed gene expression after 22 days of cold exposure, it is likely that genes involved in early cytokinin signaling were already down-regulated again. While in a cold tolerant rice cultivar the expression of the majority of the genes associated with the cytokinin signal transduction pathway was repressed, this was not true in a sensitive cultivar. This may indicate the participation of cytokinin in antagonistic cross-talk with ABA to negatively regulate cold stress tolerance in sensitive plants (Yang et al., 2015).

Furthermore, significant enrichment of cold-regulated genes was detected in the ethylene biosynthesis bin for rootstocks of both genotypes. Namely the expression of genes encoding an ethylene biosynthetic enzyme (*SGN-U579250*) and an oxidoreductase [2OG-Fe(II) oxygenase family protein (*SGN-U581679*)] was strongly decreased (log2 fold change -4.9 in LA 1777 and -4.5 in ‘Moneymaker’). This supports the hypothesis that reduced ethylene production may enhance plant tolerance to sub-optimal temperatures (Ntatsi et al., 2013), in agreement with the fact that an ethylene insensitive tomato mutant showed increased cold tolerance after chilling acclimation compared to the wild type ‘Ailsa Craig’ (Barrero-Gil et al., 2016). In contrast, in rice most ethylene biosynthesis and signaling genes were up-regulated in response to short-term cold treatment in both shoots and roots independent of the tolerance of the cultivar. In addition, some ethylene biosynthesis genes were up-regulated only in the tolerant cultivar and displayed prolonged expression during recovery (Yang et al., 2015). Since ethylene functions antagonistically with ABA, a role in detecting the relief from stress was suggested (Yang et al., 2015).

### Mainly Defense-Related Genes Are Highly Differentially Expressed between the Tolerant and Sensitive Rootstock Genotypes under Sub-Optimal Temperature in the Root Environment

The majority of the cold regulated genes that were most highly differentially expressed between the tolerant and the sensitive rootstock were involved in plant defense responses. They showed a down-regulation in LA 1777 but an up-regulation in ‘Moneymaker.’ The gene showing the highest difference between the genotypes was salicylic acid carboxyl methyltransferase, encoding an enzyme producing methyl salicylate, a mobile signal for systemic acquired resistance in plants (Park et al., 2007). Treatment of tomato fruit with methyl salicylate induces the synthesis of stress proteins, such as pathogenesis-related proteins, which lead to increased chilling tolerance and resistance to pathogens (Ding et al., 2002). Also other defense-related genes, such as genes encoding an F-box protein, two P450 cytochromes, a sigma factor binding protein and a 2-oxoglutarate-dependent dioxygenase were highly differentially cold regulated between the rootstock genotypes. When including also DEGs only identified in rootstocks of ‘Moneymaker’ but not in LA 1777 several additional pathogenesis-related genes showed a high up-regulation, e.g., genes encoding three pathogenesis related proteins with log2 fold changes of 7.1, 4.8, and 3.5, supporting the hypothesis that defense response is a major long-term cold treatment response in the sensitive rootstock. Also a comparative transcriptome analysis of cold-responsive genes in leaves of LA 1777, LA 3969, and LA 4024 identified 92 cold regulated genes with significantly different regulation between tolerant and sensitive genotypes. Stress-related GO terms, such as ‘response to stimulus’ which includes biotic stress and ‘response to stress’ were significantly enriched among these genes, together with ‘response to hormone stimulus,’ ‘response to ROS,’ and ‘calcium-mediated signaling’ (Liu et al., 2012).

Polyamine levels are increased in response to abiotic stress. They interact with ABA and ethylene to affect stress tolerance (Gill and Tuteja, 2010). Putrescine, for instance, is involved in freezing tolerance and cold acclimation in Arabidopsis (Cuevas et al., 2008) and also in the cold stress response of tomato leaves (Kim et al., 2002). The arginine decarboxylase -mediated pathway of putrescine biosynthesis is important for the cold tolerance of the tomato cultivar TNG67 and levels of putrescine and arginine decarboxylase activity are increased in both shoots and roots after cold treatment (Yang et al., 2015). In both rootstock genotypes a gene encoding the enzyme putrescine *N*-methyltransferase was down-regulated in the cold. This enzyme participates in alkaloid biosynthesis and catalyzes the biosynthesis of *S*-adenosyl-L-homocysteine and *N*-methylputrescine from *S*-adenosyl-L-methionine and putrescine. The activity of this biosynthetic pathway at the expense of putrescine could be a disadvantage under long-term sub-optimal temperature conditions when putrescine may be needed for cold adaptation. Down-regulation of this gene may therefore be advantageous for root cold tolerance.

Our subsequent and current research emphasizes to derive further relationships between genes discovered in the microarray data and appropriate physiological/biochemical processes of grafted tomato responding to suboptimal temperatures.

## Author Contributions

All authors contributed individually and meet the four criteria of authorship as required by Frontiers. In doing so, all authors agree to be accountable for the content of the work.

## Conflict of Interest Statement

The authors declare that the research was conducted in the absence of any commercial or financial relationships that could be construed as a potential conflict of interest.

## Footnotes

1 http://www.genomics.agilent.com/article.jsp?pageId=1508

2 https://solgenomics.net

3 http://www.ebi.ac.uk/arrayexpress/
