# Supplementary material for: Rootstock Sub-Optimal Temperature Tolerance Determines Transcriptomic Responses after Long-Term Root Cooling in Rootstocks and Scions of Grafted Tomato Plants
Source: Front Plant Sci. 2017 Jun 8;8:911. doi: 10.3389/fpls.2017.00911 (PMC5462977; doi:10.3389/fpls.2017.00911)
Supplement: Supplementary file 1 [file Tables_1_To_7.docx]

**Supplementary Table 1.** Effects of exposure of grafted tomato to sub-optimal and optimal temperature (T) in the root environment on total root length (L_rp_), average root diameter (2R_r_), specific root length (L_rw_) and surface area (A_rw_), and root/leaf area ratio (A_rp_/A_lp_). Grafted plants were obtained using ‘Moneymaker’ (MM) or LA 1777 (LA) as rootstock and ‘Kommeet’ (KO) as scion in the rootstock/scion (R/S) combinations. Different letters within the same column indicate significant differences between means of four replicates according to the Duncan’s Multiple Range Test (*p* ≤ 0.05). ns and * indicate nonsignificant or significant differences at P ≤ 0.05.

| Treatment | | L_rw_  (m g^-1^) | 2R_r_ (mm) | L_rp_  (m·plant^1^) | | A_rw_ (cm^2^·g^-1^) | A_rp_/A_lp_ (m^2^·m^-^²) | | |
| --- | --- | --- | --- | --- | --- | --- | --- | --- | --- |
| T | Sub-optimal | 21.9 | 0.340 a | 344 | | 168 | 0.163 | | |
|  | Optimal | 22.9 | 0.284 b | 340 | | 130 | 0.094 | | |
| R/S | LA / KO | 23.1 | 0.313 | 281 b | | 146 | 0.156 | | |
|  | MM / KO | 21.7 | 0.310 | 404 a | | 151 | 0.106 | | |
| Statistical significance | | | | | | | | | |
| T | | ns | * | ns | ns | | | * |  |
| R/S | | ns | ns | * | ns | | | * |  |
| T × R/S | | ns | ns | ns | ns | | | * |  |

**Supplementary Table 2.** Effects of exposure of grafted tomato to sub-optimal and optimal temperature (T) in the root environment on leaf net CO_2_ assimilation rate (*A*), stomatal conductance (g*_s_*), intercellular CO_2_ concentration (*c_i_*), transpiration rate (*E*), and water use efficiency (WUE). Grafted plants were obtained using ‘Moneymaker’ (MM) and LA 1777 (LA) as rootstock and ‘Kommeet’ (KO) as scion in the rootstock/scion (R/S) combinations. Different letters within the same column indicate significant differences between means of four replications according to the Duncan’s Multiple Range Test (*p* ≤ 0.05). ns, * and ** indicate nonsignificant or significant differences at P ≤ 0.05 and P ≤ 0.01, respectively.

| Treatment | | *A*  (μmol CO_2_  m^-2^ s^-1^) | | g*_s_*  (mmol  m^-2^ s^-1^) | *c*_i_  (μl l^-1^) | *E*  (mmol H_2_O  m^-2^ s^-1^) | WUE  (μmol CO_2_ mmol^-1^ H_2_O) |
| --- | --- | --- | --- | --- | --- | --- | --- |
| T | Sub-optimal | | 12.05 | 0.187 a | 273 b | 3.51 | 3.47 a |
|  | Optimal | | 10.50 | 0.158 b | 286 a | 3.43 | 3.06 b |
| R/S | LA / KO | | 11.38 | 0.165 b | 281 | 3.50 | 3.25 |
|  | MM / KO | | 11.13 | 0.180 a | 278 | 3.43 | 3.28 |
| Statistical significance | | | | | | | |
| T | | | ns | * | * | ns | ** |
| R/S | | | ns | * | ns | ns | ns |
| T × R/S | | | ns | ns | ns | ns | ns |

**Supplementary Table 3**. Effects of exposure of grafted tomato to sub-optimal and optimal temperature (T) in the root environment on maximum quantum use efficiency of PSII in the dark-adapted state (F_v_/F_m_), effective quantum use efficiency of PSII in the light-adapted state (F′_v_/F′_m_), photochemical quenching (q_P_), effective quantum yield (Φ_PSII_), non-photochemical quenching (NPQ) and steady-state fluorescence (F_s_). Grafted plants were obtained using ‘Moneymaker’ (MM) and LA 1777 (LA) as rootstock and ‘Kommeet’ (KO) as scion in the rootstock/scion (R/S) combinations. Different letters within the same column indicate significant differences between means of four replications according to the Duncan’s Multiple Range Test (*p* ≤ 0.05). ns, not significant.

| Treatment | | F_v_/F_m_ | F′_v_/F′_m_ | q_P_ | | Φ_PSII_ | NPQ | F_s_ |
| --- | --- | --- | --- | --- | --- | --- | --- | --- |
| T | Sub-optimal | 0.803 | 0.474 | 0.421 | | 0.202 | 0.593 | 2013 |
|  | Optimal | 0.797 | 0.471 | 0.372 | | 0.175 | 0.595 | 2098 |
| R/S | LA / KO | 0.798 | 0.472 | 0.395 | | 0.188 | 0.597 | 2080 |
|  | MM / KO | 0.802 | 0.474 | 0.396 | | 0.187 | 0.591 | 2031 |
| Statistical significance | | | | | | | | |
| T | | ns | ns | ns | ns | | ns | ns |
| R/S | | ns | ns | ns | ns | | ns | ns |
| T × R/S | | ns | ns | ns | ns | | ns | ns |

**Supplementary Table 4.** Effects of exposure of grafted tomato to sub-optimal and optimal temperature (T) in the root environment on glucose, fructose and sucrose in leaves and roots. Grafted plants were obtained using ‘Moneymaker’ (MM) and LA 1777 (LA) as rootstock and ‘Kommeet’ (KO) as scion in the rootstock/scion (R/S) combinations. Different letters within the same column indicate significant differences between means of four replications according to the Duncan’s Multiple Range Test (*p* ≤ 0.05). ns, not significant.

| Treatment | | Glucose | | Fructose | | Sucrose | |
| --- | --- | --- | --- | --- | --- | --- | --- |
|  |  | Leaves | Roots | Leaves | Roots | Leaves | Roots |
|  |  | (µmol mg^-1^ FM) | | | | | |
| T | Sub-optimal | 7.78 | 0.68 | 6.64 | 0.49 | 3.33 | 2.33 |
|  | Optimal | 8.45 | 0.63 | 6.82 | 0.85 | 3.08 | 2.90 |
| R/S | LA / KO | 7.36 | 0.54 | 5.84 | 0.74 | 2.98 | 2.75 |
|  | MM / KO | 8.88 | 0.76 | 7.61 | 0.60 | 3.42 | 2.48 |
| Statistical significance | | | | | | | |
| T | | ns | ns | ns | ns | ns | ns |
| R/S | | ns | ns | ns | ns | ns | ns |
| T × R/S | | ns | ns | ns | ns | ns | ns |

**Supplementary Table 5.** Effects of exposure of grafted tomato to sub-optimal and optimal temperature (T) in the root environment on guaiacol peroxidase (G-POD), glutathione Reductase (GR) and superoxide dismutase (SOD) in leaves and roots. Grafted plants were obtained using ‘Moneymaker’ (MM) and LA 1777 (LA) as rootstock and ‘Kommeet’ (KO) as scion in the rootstock/scion (R/S) combinations. Different letters within the same column indicate significant differences between means of four replications according to the Duncan’s Multiple Range Test (*p* ≤ 0.05). ns and * indicate nonsignificant or significant differences at P ≤ 0.05.

| Treatment | | G-POD | | GR | | SOD | |
| --- | --- | --- | --- | --- | --- | --- | --- |
|  |  | Leaves | Roots | Leaves | Roots | Leaves | Roots |
|  |  | (μmol mg^-1^ FM min^-1^) | | | | (Units g^-1^ FM) | |
| T | Sub-optimal | 2.82 | 3.32 a | 10.92 | 4.10 | 114 | 273 |
|  | Optimal | 2.91 | 2.32 b | 9.80 | 5.45 | 98 | 440 |
| R/S | LA / KO | 2.89 | 2.27 b | 10.14 | 3.67 | 106 | 385 |
|  | MM / KO | 2.84 | 3.37 a | 10.58 | 5.89 | 106 | 328 |
| Statistical significance | | | | | | | |
| T | | ns | * | ns | ns | ns | ns |
| R/S | | ns | * | ns | ns | ns | ns |
| T × R/S | | ns | ns | ns | ns | ns | ns |

**Supplementary Table 6.** Effects of exposure of grafted tomato to sub-optimal and optimal temperature (T) in the root environment on total amino acids in leaves and roots (AA), electrolyte leakage (EL) and starch of leaves and roots. Grafted plants were obtained using ‘Moneymaker’ (MM) and LA 1777 (LA) as rootstock and ‘Kommeet’ (KO) as scion in the rootstock/scion (R/S) combinations. Different letters within the same column indicate significant differences between means of four replications according to the Duncan’s Multiple Range Test (*p* ≤ 0.05). ns, not significant.

| Treatment | | AA | | EL  Leaves (%) | Starch | | |
| --- | --- | --- | --- | --- | --- | --- | --- |
|  | | Leaves | Roots |  | Leaves | Roots | |
|  | | (μmol mg^-1^ FM) | |  | (µmol g^-1^ FM) | | |
| T | Sub-optimal | 7.02 | 3.40 | 16.64 | 30.43 | 0.67 | |
|  | Optimal | 7.51 | 2.46 | 14.52 | 37.90 | 0.75 | |
| R/S | LA / KO | 6.60 | 3.12 | 15.49 | 33.69 | 0.74 | |
|  | MM / KO | 7.93 | 2.82 | 15.67 | 35.05 | 0.69 | |
| Statistical significance | | | | | | |  |
| T | | ns | ns | ns | ns | ns | |
| R/S | | ns | ns | ns | ns | ns | |
| T × R/S | | ns | ns | ns | ns | ns | |

**Supplementary Table 7.** Effects of exposure of grafted tomato to sub-optimal and optimal temperature (T) in the root environment on lipid peroxidation (MDA), hydrogen peroxide (H_2_O_2_) and total proteins of leaves and roots. Grafted plants were obtained using ‘Moneymaker’ (MM) and LA 1777 (LA) as rootstock and ‘Kommeet’ (KO) as scion in the rootstock/scion (R/S) combinations. Different letters within the same column indicate significant differences between means of four replications according to the Duncan’s Multiple Range Test (*p* ≤ 0.05). ns and * indicate nonsignificant or significant differences at P ≤ 0.05.

| Treatment | | MDA | | H_2_O_2_ | | Protein | | |
| --- | --- | --- | --- | --- | --- | --- | --- | --- |
|  |  | Leaves | Roots | Leaves | Roots | Leaves | | Roots |
|  |  | (nmol g ^-1^ FM) | | (μmol g^-1^ FM) | | (mg g^-1^ FM) | | |
| Sub-optimal T | LA / KO | 8.77 | 4.79 | 25.45 | 3.17 | 3.15 ab | 0.200 | |
|  | MO / KO | 8.85 | 2.67 | 14.17 | 3.40 | 3.85 a | 0.618 | |
| Optimal T | LA / KO | 7.86 | 3.61 | 14.00 | 3.90 | 3.73 a | 0.835 | |
|  | MM / KO | 6.63 | 2.54 | 22.65 | 3.41 | 1.68 b | 2.205 | |
| Statistical significance | | | | | | | | |
| T | | ns | ns | ns | ns | ns | | ns |
| R/S | | ns | ns | ns | ns | ns | | ns |
| T × R/S | | ns | ns | ns | ns | * | | ns |
